# Ion-Pairing Chromatography and Amine Derivatization Provide Complementary Approaches for the Targeted LC-MS Analysis of the Polar Metabolome

**DOI:** 10.1021/acs.jproteome.2c00030

**Published:** 2022-05-10

**Authors:** Virag Sagi-Kiss, Yufeng Li, Matthew R. Carey, Sarah J. Grover, Karsten Siems, Francesca Cirulli, Alessandra Berry, Chiara Musillo, Ian D. Wilson, Elizabeth J. Want, Jacob G. Bundy

**Affiliations:** † Department of Metabolism, Digestion and Reproduction, Imperial College London, South Kensington, London SW7 2AZ, U.K.; ‡ AnalytiCon Discovery GmbH, Hermannswerder Haus 17, 14473 Potsdam, Germany; § Center for Behavioral Sciences and Mental Health, Istituto Superiore di Sanità, Viale Regina Elena, 00161 Rome, Italy; ∥ Department of Psychology, Sapienza University of Rome, via dei Marsi 78, 00185 Rome, Italy

**Keywords:** metabolomics, metabonomics, ion-pairing, ampelopsin, healthy aging, UPLC-MS, NMR spectroscopy, statistical heterospectroscopy

## Abstract

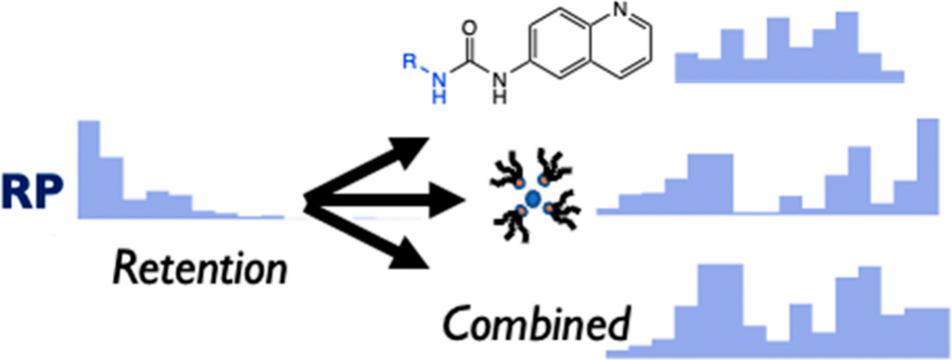

Liquid chromatography coupled to mass spectrometry is a key metabolomics/metabonomics technology. Reversed-phase liquid chromatography (RPLC) is very widely used as a separation step, but typically has poor retention of highly polar metabolites. Here, we evaluated the combination of two alternative methods for improving retention of polar metabolites based on 6-aminoquinoloyl-*N*-hydroxysuccinidimyl carbamate derivatization for amine groups, and ion-pairing chromatography (IPC) using tributylamine as an ion-pairing agent to retain acids. We compared both of these methods to RPLC and also to each other, for targeted analysis using a triple-quadrupole mass spectrometer, applied to a library of ca. 500 polar metabolites. IPC and derivatization were complementary in terms of their coverage: combined, they improved the proportion of metabolites with good retention to 91%, compared to just 39% for RPLC alone. The combined method was assessed by analyzing a set of liver extracts from aged male and female mice that had been treated with the polyphenol compound ampelopsin. Not only were a number of significantly changed metabolites detected, but also it could be shown that there was a clear interaction between ampelopsin treatment and sex, in that the direction of metabolite change was opposite for males and females.

## Introduction

Metabolomics/metabonomics, as a scientific field, depends on the analytical ability to profile metabolites from a wide range of sample types. There are many approaches to metabolite profiling, but the vast majority of published papers use either nuclear magnetic resonance (NMR) spectroscopy or mass spectrometry (MS) as analytical platforms. ^1^H NMR is most commonly used to analyze complex mixtures directly; MS is frequently hyphenated to a separation technique, of which the two most common are gas and liquid chromatography (GC and LC). All of these techniques have their own specific advantages and disadvantages: NMR is unmatched as a universal and quantitative untargeted detector,^1−3^ but the high mass requirement means that it is generally limited to detection of the highest concentration metabolites only. GC is the most natural separation partner to MS, as the analytes are already in the gas phase in the separation step, and furthermore it offers excellent chromatographic performance; but it is generally necessary to derivatize metabolites to make them volatile, and it is limited in its coverage of key metabolite groups. LC-MS has the potential to offer the widest coverage of the metabolome, although there are also some important limitations. Critically, the separation step is potentially limiting.^4^

The “standard” LC separation technique is reversed-phase (RP) chromatography, which uses a polar mobile phase (prototypically, water/methanol or water/acetonitrile) and a nonpolar stationary phase (prototypically, C18—octadecyl-bonded silica). The term “standard” should be used with caution, as there are a plethora of different phases and supports available from different manufacturers, which may offer useful variation in retention characteristics—nonetheless, there is sufficient commonality that they can be considered as a group. There are many reasons why RPLC is so widely used as a separation method: it provides a robust and reproducible platform, the retention characteristics are understandable and predictable, and it is compatible with aqueous biological samples. It is generally the method of choice for nonpolar or semipolar metabolites. However, highly polar metabolites are more problematic, as they have only poor retention, eluting shortly after the void volume. These include some of the most biologically important metabolites, which are critical to all kinds of studies. Even if there is some retention for such analytes, significant ion suppression can be expected, and it is certainly suboptimal.

There are a number of approaches which are, or should be, complementary to RPLC for metabolome profiling. In particular, hydrophilic liquid interaction chromatography (HILIC) is very widely used for metabolomics.^5−7^ There are, however, also limitations to HILIC. For example, analyte peaks may be broader and less Gaussian than for RPLC; retention time shifting can potentially be an issue, which has to be mitigated via long re-equilibration times; and samples are generally redissolved in a high concentration of organic solvent for injection, which can lead to solubility problems. There is a clear need for additional development of LC methods that improve retention of polar metabolites and also enable the analysis of metabolites that are not well suited to HILIC (e.g., see ref (8)).

One set of methods makes use of the beneficial properties of RPLC by modifying metabolites to improve their retention—either permanently, by chemical derivatization, or temporarily, by adding modifiers to the mobile phase. Ion-pairing chromatography (IPC) mixes amphiphilic molecules with the phase—for instance, a positively charged surfactant molecule would be suitable for negatively charged analytes, as it would form ion-pairs with anions, which would then be retained by RP mechanisms.^9^ Of course, when using MS as a detector, there is an additional complicating factor that the ion-pairing agents should be sufficiently volatile to be compatible with the mass spectrometer. Alkyl amines are often used for IPC of anionic analytes, as they are more volatile than more strongly surface-active compounds such as quaternary alkyl ammonium compounds, and their charge can be controlled by adjusting the mobile phase pH. Because of the improvement of retention achieved through IPC, a number of different studies have applied IPC to improve metabolome coverage and analytical methods.^8,10−15^

An alternative to IPC is covalent modification of analytes by derivatization. This is, of course, a substantial area of research;^16,17^ we merely note here that covalent derivatization methods have a long history in chromatography. 6-Aminoquinoloyl-*N*-hydroxysuccinidimyl carbamate was originally developed for amino acid analysis using optical detection (both fluorescence and absorbance),^18,19^ but was later adopted for use with mass spectrometric detection, opening up the potential for using it for the broad analysis of the amine-containing submetabolome.^20^ Gray et al. monitored 66 potential analytes and quantified 25 in human plasma samples obtained from patients with varying degrees of liver failure.^33^

Here, we systematically evaluate the combination of two methods that have both been previously used independently for polar metabolite analysis: derivatization of amines by 6-aminoquinoloyl-*N*-hydroxysuccinidimyl carbamate, and ion-pairing using tributylamine. The capabilities of these methods were explored using a large library of standards, and also by application to the real-world analysis of biological samples.

## Materials and Methods

### Chemicals and Reagents

The mass spectrometry metabolite library (MSMLS) was from IROA Technologies (NJ, USA). Other chemical standards not in the MSMLS library, formic acid (FA), chloroform (CHCl_3_), acetonitrile (ACN), deuterium oxide (D_2_O, tributylamine (TBA), acetylacetone (AAc), acetic acid (HAc), sodium phosphate monobasic and dibasic, D_2_O, and isotopically labeled internal standard, l-phenyl-*d*_5_-alanine, were obtained from Sigma-Aldrich (Gillingham, U.K.). AccQTag Ultra reagent was obtained from Waters UK (Wilsmlow, UK). LC-MS grade water, water with 0.1% FA (v/v), and ACN with 0.1% FA (v/v) were purchased from Fisher Scientific (Leicester, U.K.). Methanol (MeOH) and isopropanol (IPA, LC-MS grade) were obtained from Honeywell (Charlotte, NC, U.S.A.). Sodium trimethylsilylpropanesulfonate solution (DSS-*d*_6_, IS-2) was obtained from Chenomx (Alberta, Edmonton, Canada).

### Mouse Experiments

The experimental subjects were 24 male and 26 female mice, aged from 18 to 20 months, of the C57BL/6N strain. Same sex conspecifics were housed 4 to 5 per cage and treated with 1% ampelopsin (10 g/kg of food) pellet food or via a control diet (same composition but without ampelopsin). Ampelopsin was provided from AnalytiCon Discovery (Hermannswerder Haus 17, 14473 Potsdam, Germany) and both the control and the ampelopsin diets were custom-made by Ssniff Spezialdiäten (Ferdinand-Gabriel-Weg 16, D-59494 Soest, Germany). A special low-antioxidant diet was used (depleted in vitamins C and E, and low in phytoestrogens) in order to maximize any potential antioxidant effect of ampelopsin. All subjects were sacrificed by decapitation. All peripheral and central tissues were rapidly dissected and snap frozen in liquid nitrogen for further analyses. All experimental procedures were reviewed by the ethical body of the Istituto Superiore di Sanità for animal welfare and conducted in conformity with the European Directive 2010/63/EU and the Italian legislation on animal experimentation, D. Lgs. 26/2014. They were authorized by the Italian Ministry of Health.

### Sample Handling

The samples were extracted and analyzed in a randomized block design, to avoid any potential confounding of the experimental factors with the running order. All samples were anonymized during analysis, and tracked using alphanumeric codes generated using cual-id software.^21^

### Tissue Extraction

Liver samples were extracted following a modification of the classic Bligh and Dyer approach for lipid extraction.^22^ One male ampelopsin-treated sample was lost during extraction. Samples were kept frozen on dry ice and extracted in random block order in order to minimize any bias. The frozen tissue was added to prechilled 7 mL bead beater tubes containing 1.4 mm zirconia beads. Samples had cold (−20 °C) MeOH/CHCl_3_ volume adjusted based on weight, with 0.3 mL added per 100 mg tissue in a 2:1 ratio for MeOH:CHCl_3_. Samples were processed from frozen in a Precellys Evolution bead beater (Stretton Scientific, Stretton, UK) at 10 000 rpm for 20 s. An additional 0.1 mL each of water and of CHCl_3_ per 100 mg tissue was then added to separate the phases, and the samples were then mixed in the bead beater (10 s, 4500 rpm) and then centrifuged (3000*g*, 10 min.). 500 μL of the upper aqueous layer was removed and dried overnight at 30 °C using a vacuum concentrator.

### Metabolite Library

The MSMLS library was manually edited to remove mislabeled and duplicate metabolites. Metabolite standards were made in H_2_O or H_2_O/MeOH mixture to a final concentration of typically 10 μg/mL and stored at −80 °C. Further dilutions were always made with H_2_O. For direct infusion single standards were made up at 1 mg/mL and diluted with water. Mixtures of 12 compounds (with different masses at unit resolution) were pooled to determine retention time/parent ion/fragment ion (*t*_R_/Q1/Q3) data for compound identification. Parent ions and fragments were determined from the XCMS-MRM database^23^ where possible; the database collision energy (CE) was converted to a predicted value for the XEVO-TQS based on the behavior of a number of experimental CE values for standards compared to the database values. The best CE was then determined by ramping around the predicted value in increments of 2–5 V. Those compounds with positive molecular ions in XCMS-MRM were only tested with positive mode RPLC and (where appropriate) the AccQ-Tag derivatization method.

Compounds that were not present in the XCMS-MRM database, or those for which we failed to obtain *t*_R_/Q1/Q3 values using the above step, were directly infused to the MS and the vendor built-in optimization process was used to determine best CE and best Q1 and Q3 values. The *t*_R_ of this group of compounds was then determined in a second LC run.

For AccQ-Tag derivatized standards, [M + 171]^+^ was the observed parent ion for monoamines and CE was optimized for the highest abundance fragment ion (171.05). For compounds with two or more amine groups, the maximum number of AccQ-Tag additions and charges was used to define the parent ion, but other derivatization products^20^ were also recorded and the relevant *t*_R_/Q1/Q3 values added to the database to assist annotation in real samples by helping to identify potential interferences.

### Derivatization

Standards and samples were derivatized according to the AccQ-Tag Ultra Kit (Waters UK Ltd., Wilmslow, UK) derivatization procedure; briefly, 10 μL sample were mixed with 70 μL borate buffer and 20 μL AccQ-Tag reagent. After a few minutes at room temperature samples were heated to 55 °C for 10 min to degrade the excess AccQ-Tag reagent. Samples were then diluted 1:5 with water while standards were diluted between 10 and 100 times as appropriate. Further dilutions were carried out for analysis of samples if the chromatographic peaks were observed to saturate the MS detector.

### Biological Samples

These were analyzed using the same analytical procedures as given above. Phenylalanine-^2^H_5_ was included as an injection standard, in order to check injection volume stability, but was not used to normalize the data. The freeze-dried samples were reconstituted in 100 μL H_2_O, and centrifuged (16 000*g*, 10 min). The reconstituted solution was directly injected for ion pairing chromatography, and a 10 μL aliquot was frozen (if not being used immediately) and stored until derivatization. Blanks (both process blanks and reagent blanks) and quality control (QC) samples, consisting of a pooled equal volume of all samples, were also analyzed; the QC samples included a run of 5 samples before and after the main run, and then every 10th sample during the run was a QC sample.^24,25^ The QCs also provide a type of system suitability test, along with the normal prerun calibration and testing of the MS: the run was not started unless the prerun QCs showed evidence of stabilization of *t*_R_ and peak shape.

### UHPLC-MS Settings

For UHPLC-MS analysis 5 μL of sample was injected with UHPLC separations performed on 2.1. mm i.d. columns of either 100 (RPLC, IPC) or 150 mm (AccQ-Tag RPLC) in length packed with the 1.8 μm C18 bonded HSS T3 stationary phase (Waters). The mobile phases were either (A) 0.1% FA in H_2_O (AccQ-Tag, RPLC) or TBA:HAc:AAc in H_2_O (IPC) and (B) 0.1% FA in ACN (AccQ-Tag, RP) or MeOH:IPA (IPC), using flow rates of 0.4 (RPLC, IPC) or 0.6 mL/min (AccQ-Tag RPLC). Gradient conditions were optimized for each method and provided as Table S1, Supporting Information). Each metabolite was scheduled within a 1 min window of the measured *t*_R_ (i.e., ± 30s). For all methods, a binary solvent manager, sample manager, and column manager (Waters, Milford, MA, U.S.A.) interfaced to a Xevo TQ-S tandem quadrupole mass spectrometer (Waters. Corp) was used. A dedicated instrument was used for IPC, as otherwise the ion pairing molecules cause contamination in other samples. Details of ionization conditions, gas flows, etc. can be found in Table S1. The analysis times for the RPLC, IPC, and AccQ-Tag RPLC methods were 14.5, 21.0, and 13.0 min, respectively. Acetylacetone was included in the mobile phase for the ion-pairing chromatography, as it has been shown to improve analytical performance for similar samples.^26^

### Data Processing

The data were processed using the freeware package Skyline.^27^ Metabolite assignment was based on matching retention time, ion ratios, and peak shape comparison between samples and authentic standards, plus absence of signals from blanks. QC samples with authentic standards spiked in were used in some cases to assist peak annotation. Known interferences from in-source fragments were included in the workflow used for peak annotation (e.g., but not limited to, ATP for ADP signal, UDP-glucose for UDP, adenosine for adenine, malate for fumarate, citrulline for ornithine, etc.). Relative standard deviations (RSDs) were calculated using the QC samples, as described above.

### NMR Spectroscopy

Metabolite profiling by ^1^H NMR was carried out using a Bruker Avance DRX600 spectrometer, operating at 600 MHz and equipped with a 5 mm inverse probe. Samples were introduced using a SampleJet autosampler; they were cooled at 4 °C before acquisition and kept at 25 °C during acquisition. The samples were dissolved in 0.65 mL of NMR buffer (phosphate buffer, pH 7, 0.2 M; 0.1 mM DSS-*d*_6_; made up in D_2_O), centrifuged (5 min, 16 000*g*), and 0.6 mL transferred into 5 mm SampleJet tubes. 1D spectra were acquired using an automation sequence that performed tuning and matching, shimming, and measurement of 90° pulse length on each individual sample.^28^ The data were then acquired using a NOESYPRESAT sequence for water suppression, with 64 scans and 8 dummy scans per sample. The data were acquired into 20 ppm spectral width and 64 K data points, giving an acquisition time of 2.3 s; an additional relaxation delay of 2.7 s was used to give an overall recycle time of approximately 5 s.

The spectra were processed with a 0.3 Hz exponential apodization function; automated algorithms were used to adjust phase, baseline, and reference chemical shift to DSS (δ = 0). The processed spectra were then opened in NMR Suite 8 (Chenomx, Edmonton, Canada), and manual metabolite deconvolution was performed. Metabolite assignment was made on the basis of 2D NMR spectra as well as the 1D spectra used for profiling; four metabolites (inosine, adenosine, uridine, and hypotaurine) had their identities further confirmed by spiking experiments.

### Data Analysis

All data were normalized using the probabilistic quotient method.^24^ The data were then analyzed by *t* tests for two-group comparisons (i.e., ampelopsin-treated vs control for the male and female mice separately), and by two-way analysis of variance with “sex” and “ampelopsin treatment” as factors. Principal component analysis used data that had been mean-centered and transformed to unit variance. Statistical significance was evaluated at *P* < 0.01, and Bonferroni correction was used where multiple tests were carried out.

## Results and Discussion

We have used a targeted approach in the current study, i.e., profiling only known metabolites. Targeted metabolomics is a separate, although closely related, field to untargeted metabolomic analysis: it is not our intent to claim superiority for one over the other, but only to point out that both can be used as valid approaches to biochemical exploration.^29,30^

Other studies have also used targeted or pseudotargeted methods to give coverage of a wide range of metabolites,^31^ including ones based on the same metabolite library that we have used here.^32^ However, we have combined different separation methods with greater metabolite library coverage than has been reported previously.

We tested 111 amine metabolites by derivatization with AccQ-Tag Ultra reagent (a commercially available kit for derivatization by 6-aminoquinoloyl-*N*-hydroxysuccinidimyl carbamate). The chromatographic performance of the derivatized analytes was excellent, as others have also found:^33^ the peaks were dispersed well over the full width of the chromatogram, and a variety of critical pairs (e.g., leucine and isoleucine) were separated. It should be noted that care must be taken with metabolites with multiple amine groups, as these will form multiple derivatives. However, when such multiple derivatives were formed they were minor, typically accounting for less than (1% by response) of the major product.

**Figure 1:**
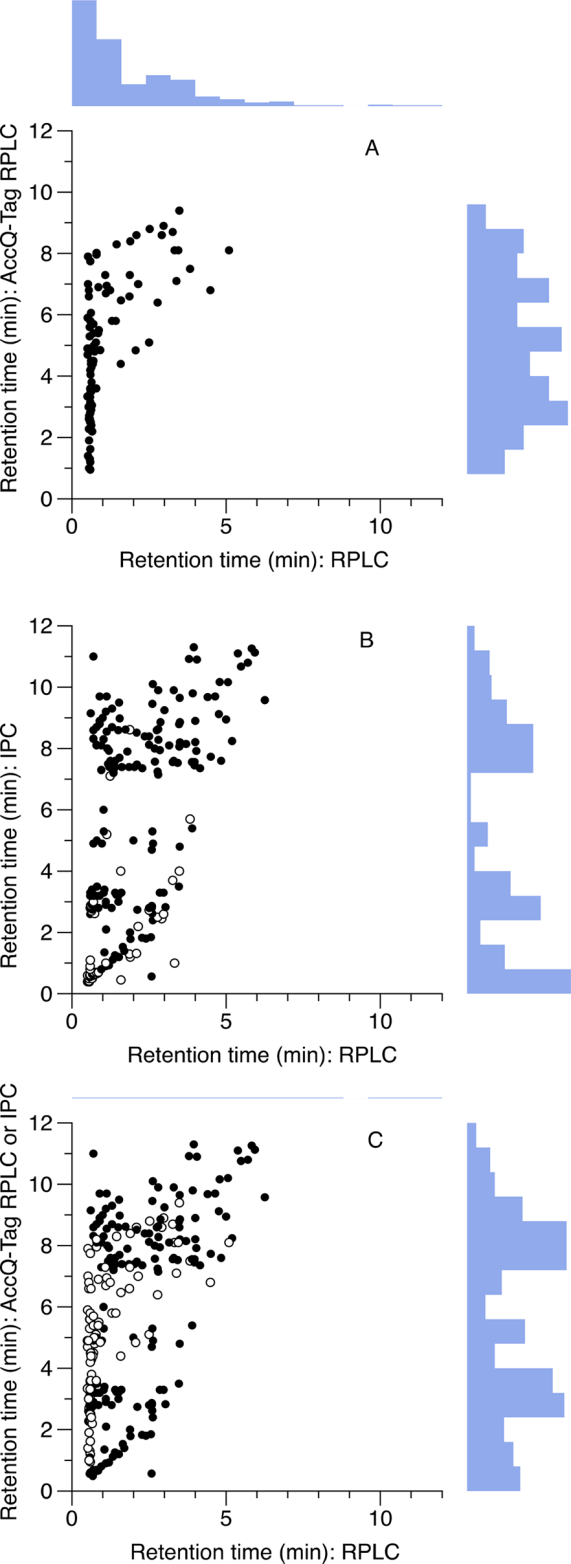
Retention times (*t*_R_) of polar metabolites are improved both by IPC or AccQ-Tag derivatization, and the combination of both approaches together is highly complementary. (A) RPLC compared to AccQ-Tag RPLC. (B) RPLC compared to IPC. Compounds which were also detected by AccQ-Tag RPLC are shown by open symbols. (C) RP compared to IPC (filled symbols) and AccQ-Tag (open symbols) as a combined strategy; where a metabolite can be analyzed by either technique, the AccQ-Tag data are shown. Histograms show distribution of retention time data.

This confers a substantial real-world benefit when compared to RPLC for the same analytes: 102 metabolites were detected by both AccQ-Tag RPLC and RPLC, and for these, all metabolites had their *t*_R_ increased compared to the equivalent RPLC value (Figure 1). In particular, 74 of these 102 metabolites had unacceptable retention for RPLC (*t*_R_ < 1.0 min), and a further 7 had borderline retention characteristics (1.0 min < *t*_R_ < 1.5 min; Figure 1).

A handful of metabolites had poor retention by both methods (*t*_R_ < 1.0 for RPLC and *t*_R_ < 1.5 for AccQ-Tag RPLC)—principally the sugar amines and related compounds (glucosamine, galactosamine, mannosamine, glucosaminic acid, glucosamine-6-phosphate, glucosamine-6-sulfate), but also histidinol. The sugar amine compounds also gave rise to multiple peaks; because of this, and because the sugar amines are not resolved at unit mass, we have not reported data from these compounds for biological mixtures. In general, we recommend against analysis of the sugar amines by AccQ-Tag derivatization, unless extra care is taken (e.g., by injecting authentic standards spiked into actual samples). However, the sugar amine derivatives glucosaminic acid, glucosamine-6-sulfate, and glucosamine-6-phosphate, could be clearly separated and identified in biological samples.

The IPC method was less uniformly good in terms of peak shape: thus, while many compounds gave excellent Gaussian peaks, some provided broadened or asymmetrical peaks (see Supplementary Figure S1). We also compared two different ion-pairing reagents; diisopropylethylamine, with hexafluoroisopropanol as a weak acid modifier. The latter has been suggested as offering potentially greater sensitivity compared to tributylamine/acetic acid.^34^ Our preliminary analysis, using a selected number of compounds, showed a slight advantage in sensitivity; however, it performed substantially worse in metabolite retention, and so we continued with tributylamine/acetic acid only (Supplementary Figure S2).

We successfully measured *t*_R_ for 283 of the metabolites by IPC. Where *t*_R_ was not obtained, this was due either to low sensitivity of the metabolite in negative ESI, or else poor chromatographic performance. Of these metabolites, 244 also had a *t*_R_ successfully assigned by RPLC. The *t*_R_ data for all metabolites are given in the Supporting Information, Table S2. In general, however, IPC was very effective at improving the retention of otherwise poorly retained metabolites. Only three metabolites had clearly greater retention for RP than IPC: dopamine, tryptophanamide, and TRH. (The first two were well retained by AccQ-Tag RPLC, so in practice, would be preferentially analyzed by this method rather than either IPC or RPLC.) Overall, RPLC had poor performance, when judging the retention of polar metabolites: 40% (98 of 243) of the jointly detected metabolites had an unacceptable *t*_R_ < 1.0, and a further 15% (37 metabolites) fell in a borderline category of *t*_R_ < 1.5 min. In contrast, IPC had only 21% of metabolites (60 out of 283) with unacceptable *t*_R_ < 1.0 min, and a further 5% (14 out of 283) with borderline *t*_R_ < 1.5 min (Figure 1). Interestingly, the IPC *t*_R_ data appeared to have an approximately bimodal distribution, with “peaks” around 2–3 and 8–9 min.

The two methods for polar metabolite retention, IPC and AccQ-Tag RPLC, were only weakly associated with respect to retention characteristics (*r*^2^ = 0.12, 57 metabolites with data for both techniques). This is advantageous when it comes to combining methods. If both IPC and AccQ-Tag RPLC are used, 334 potential analytes can be determined; however, if the *t*_R_ data are also compared to RPLC, this reduces the number to 287 metabolites. By combining AccQ-Tag RPLC with an IPC analysis, metabolite coverage was improved from ca. 40% with good retention (*t*_R_ > 1.5 min) by RPLC to ca. 90% (Figure 1). The remaining metabolites, which were not retained well by any method here, include, unsurprisingly, sugars and polyols (trehalose, raffinose, stachyose, galactitol, erythritol, and xylitol; other common sugar metabolites were not included here, precisely because they are notoriously problematic analytes for LC-MS, but we can nonetheless safely conclude that our combined method is not suitable for sugars or polyols) and a number of other small and highly polar metabolites, e.g., nucleobases.

Another key factor for any analytical method is sensitivity—at what concentration can we detect specific metabolites? We did not aim to characterize the whole metabolite library, but instead compared a small number of representative metabolites between the RPLC and IPC methods. We picked 7 metabolites, including basic, acidic, and lipophilic amino acids (Gln, Glu, Phe, Trp), an acid (citrate), a nucleoside and a nucleotide (cytidine, GMP), and evaluated the response on the same mass spectrometer, i.e., keeping all of the parameters as comparable as possible except for the chromatography. We did not attempt to calculate formal limits of detection but compared peak areas to give a broad indication of any major effects on signal intensity. The effects were small and showed little clear trend toward either increased or decreased sensitivity in the IPC-MS compared to the RPLC-MS: the difference in sensitivity for RPLC compared to IPC ranged from a 4 fold increase (Trp) to a 0.7 fold decrease (Gln), with a median fold change difference of only 1.1 fold (Supporting Information, Figure S3). This is, admittedly, only a small number of metabolites, but does at least suggest that the inclusion of the ion-pairing reagent is not dramatically and universally reducing the sensitivity of analytes across the board.

We tested our combined method by analyzing a set of real biological samples: liver extracts from aged mice that had been treated with a polyphenol compound, ampelopsin, with potential healthspan benefits.^35−37^ In total, for the IPC-MS, we detected 193 transitions from 85 metabolites. We imputed any missing values by replacing them with half the minimum value observed for that metabolite. For the AccQ-Tag data, we observed 75 transitions from 72 metabolites (NB that while almost all metabolites had the single daughter ion *m*/*z* = 171, derived from the derivatized group, cysteate had 3 and *N*-acetyllysine had 2 transitions monitored, respectively). The data for the metabolites monitored in our final method (including *t*_R_, detection mode, parent and daughter ions, and collision energy) are given in Supplementary Table S3.

**Figure 2:**
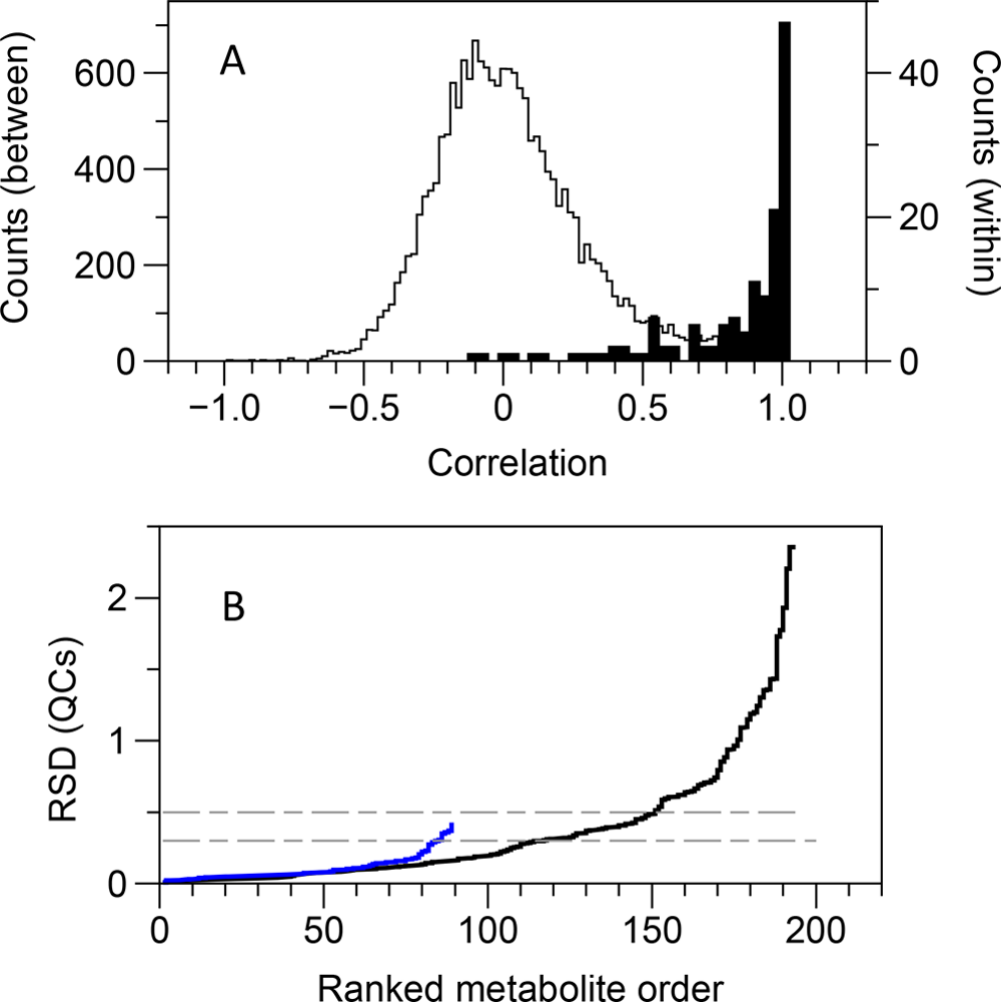
Reproducibility of data for real biological samples (liver extracts). (A) Within-metabolite correlations (i.e., multiple transitions per metabolite for the ion-pairing data; black histogram) are much higher than the between-metabolite transitions (gray histogram). (B) Cumulative distributions of relative standard deviation for pooled quality-control samples. Blue line: AccQ-Tag RPLC. Black line: ion-pairing chromatography. The dashed horizontal gray lines indicate RSD cutoffs of 0.3 (AccQ-Tag RPLC) and 0.5 (IPC).

For the IPC data, we analyzed all the transitions separately, as opposed to selecting a single best transition for each metabolite. Both approaches are defensible: by analyzing all transitions, we do not prejudge which is the best, and, where more than one transition is present for a single metabolite, similar statistical behavior of the variables helps to validate individual metabolites in a simple and straightforward way. There were 193 transitions (after manual processing and assessment of the data in Skyline—i.e., compounds which were clearly absent or of very poor quality were already excluded). Of these, 60% had a relative standard deviation (RSD) < 0.3, and 78% had a RSD < 0.5 (Figure 2). The choice of a threshold is, of course, arbitrary, although 0.3 has been widely used for metabolomic data.^25^ For the current data set, if the RSD_QC_ is plotted against the ratio of the RSD of the biological samples to the QC samples (RSD_biol_/RSD_QC_), there is an apparent step in the data above RSD_QC_ = 0.5 (Supplementary Figure S4), and so we would suggest that RSD_QC_ < 0.5 is appropriate for finding potentially interesting metabolites in this specific IPC-MS data set. This approach gave either 58 or 69 metabolites for the two QC thresholds, respectively. Nine of these were also present in the AccQ-Tag RPLC data, so in total—AccQ-Tag RPLC plus IPC—we detected 132 metabolites with confidence in the liver extracts by LC-MS.

Another method of assessing the quality of the data is to look at the correlation between the different transitions of a metabolite, where more than one transition has been assigned. We have not done this for the AccQ-Tag data, as here we monitored a single fragment (*m*/*z* = 171), with only a couple of exceptions. For the IPC data, the nonstructural correlations (i.e., between all metabolites, and considering all 193 transitions) had, as expected, a broad, symmetrical distribution around *r* = 0; conversely, the structural correlations (within metabolites) had an extremely right-skewed distribution, where about half (73 out of 138 correlations) had *r* > 0.95 (Figure 2). The two distributions were well discriminated (area under ROC curve = 0.97).

Finally, in terms of data validation, we also acquired data for these samples by 1D ^1^H NMR spectroscopy. We assigned and fitted 30 metabolites from these data, using a commercial software package for computer-assisted manual fitting. We have previously shown that manual fitting is reproducible, highly so if a single individual performs the fitting,^38^ as was the case here. Fourteen of the metabolites were not observed in either the IPC or the AccQ-tag data, and so in total, across all three platforms, we detected 146 metabolites. The data from the AccQ-Tag, ^1^H NMR spectroscopy, and IPC-MS are given in the Supporting Information, Tables S4, S5, and S6, respectively.

Metabolites that were detected by more than one method can be directly cross-compared across all samples, an approach termed statistical heterospectroscopy (SHY).^39^ SHY can be used not only for potential assignment of unknowns, but also to increase confidence in the assignment of known compounds.^40^ Fifteen metabolites were detected in common between the NMR spectroscopic and both of the LC-MS data sets. (We considered the NMR data set to be our validating data set, given the excellent reproducibility of NMR spectroscopic data, and so we did not examine correlations between the two MS data sets.) Overall, there was a very clear discrimination between structural and nonstructural correlations (*P* = 2 × 10^–9^, logistic regression; AUROC = 0.87). Some metabolites (e.g., 2-aminoadipate) were detected on all three platforms, and showed excellent correlation across all of them (Supporting Information, Figure S5).

**Figure 3:**
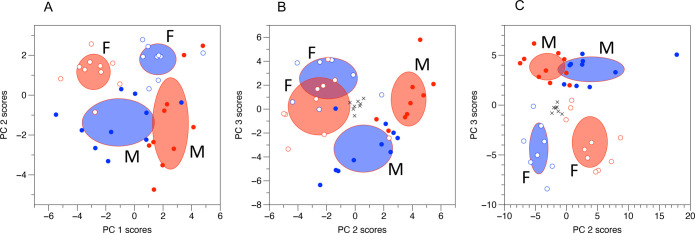
Multivariate analysis of metabolomic data indicates that there is an effect of sex, but that ampelopsin manifests as an interaction with sex, with opposing metabolic effects in male and female mice. Principal components analysis: empty symbols = females, filled symbols = males; red = controls, blue = ampelopsin treated mice. Black crosses indicate quality control samples. Ellipses represent ± SD; M and F label the SD ellipses for male and female mice, respectively. (A) ^1^H NMR spectroscopic data; (B) AccQ-Tag RPLC data; (C) IPC data.

Multivariate analysis (principal components analysis, PCA) showed essentially the same biological picture for all three of the data sets. The values from the QC samples were tightly clustered in the center of the plot for both of the LC-MS data sets, demonstrating the high technical reproducibility of the data. There was a strong metabolic difference between the male and female mice, and there was no clear metabolic effect of ampelopsin alone, but there was a strong interaction with sex; i.e., ampelopsin had opposite effects in the male and female mice (Figure 3). The NMR spectroscopic data had a significant effect of sex along PC 2, and a significant interaction along PC 1; the AccQ-Tag RPLC data showed a similar pattern across PCs 2 and 3, but not aligned with the axes, so that both sex and interaction were significant on both PCs; and the IPC data had a significant effect of sex along PC 3, and a significant interaction along PC 2 (Table 1).

**Table 1:** Significance of Principal Component Scores with Respect to the Experimental Factors “Sex” And “Ampelopsin Treatment” for Three Different Data Types

	principal component	sex	ampelopsin	interaction
^1^H NMR	1	0.20	0.43	6.1 × 10^–9^
	2	3.7 × 10^–7^	0.46	0.55
	3	0.031	0.27	0.78
AccQ-Tag RPLC	1	0.34	0.77	0.15
	2	7.4 × 10^–8^	0.055	0.0048
	3	0.00067	0.21	5.5 × 10^–5^
IPC	1	0.56	0.85	0.024
	2	0.5	0.62	1.2 × 10^–7^
	3	1.1 × 10^–13^	0.49	0.77

**Figure 4:**
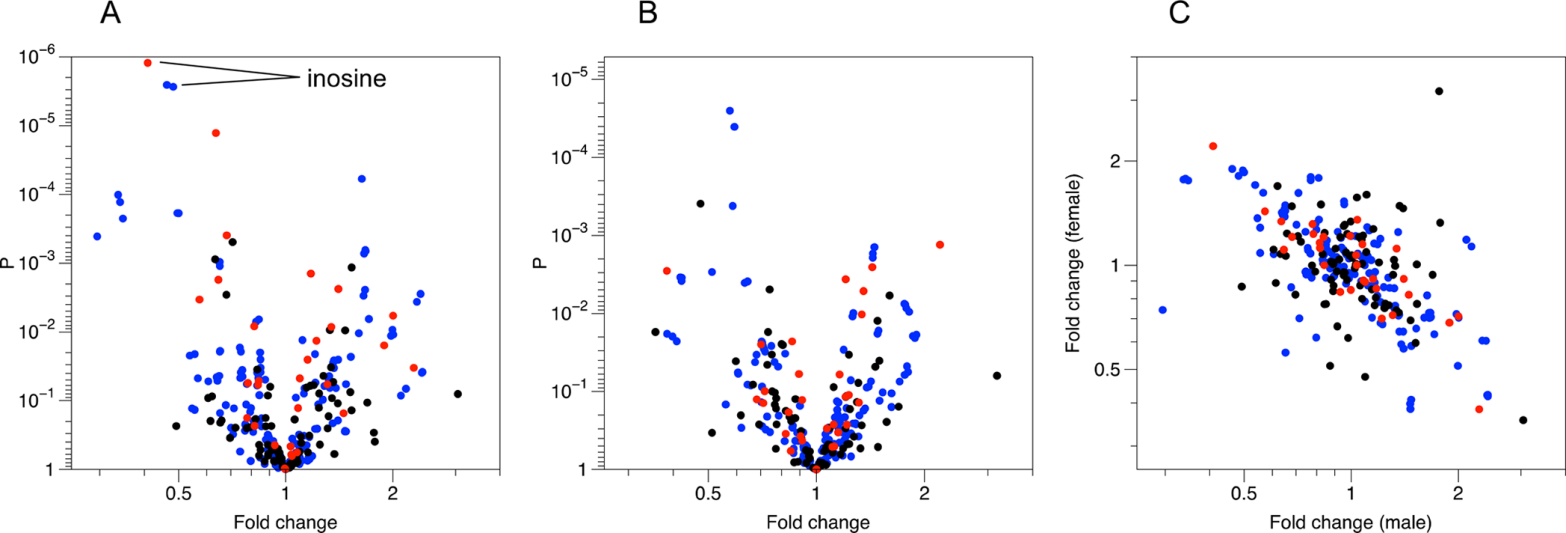
Univariate analyses identify metabolites with high significance for ampelopsin treatment in both male and female mice, and the effects tend to be opposite in males and females. Red: ^1^H NMR data; blue: IPC data; black: AccQ-Tag RPLC data. (A) Volcano plot for male mice. One metabolite, inosine, is annotated as an example, identified by three different variables: one ^1^H NMR spectroscopic measurement, and two transitions from the LC-MS ion pairing data. (B) Volcano plot for female mice. (C) Fold change values for males against females show a negative correlation across all three analytical platforms.

**Figure 5:**
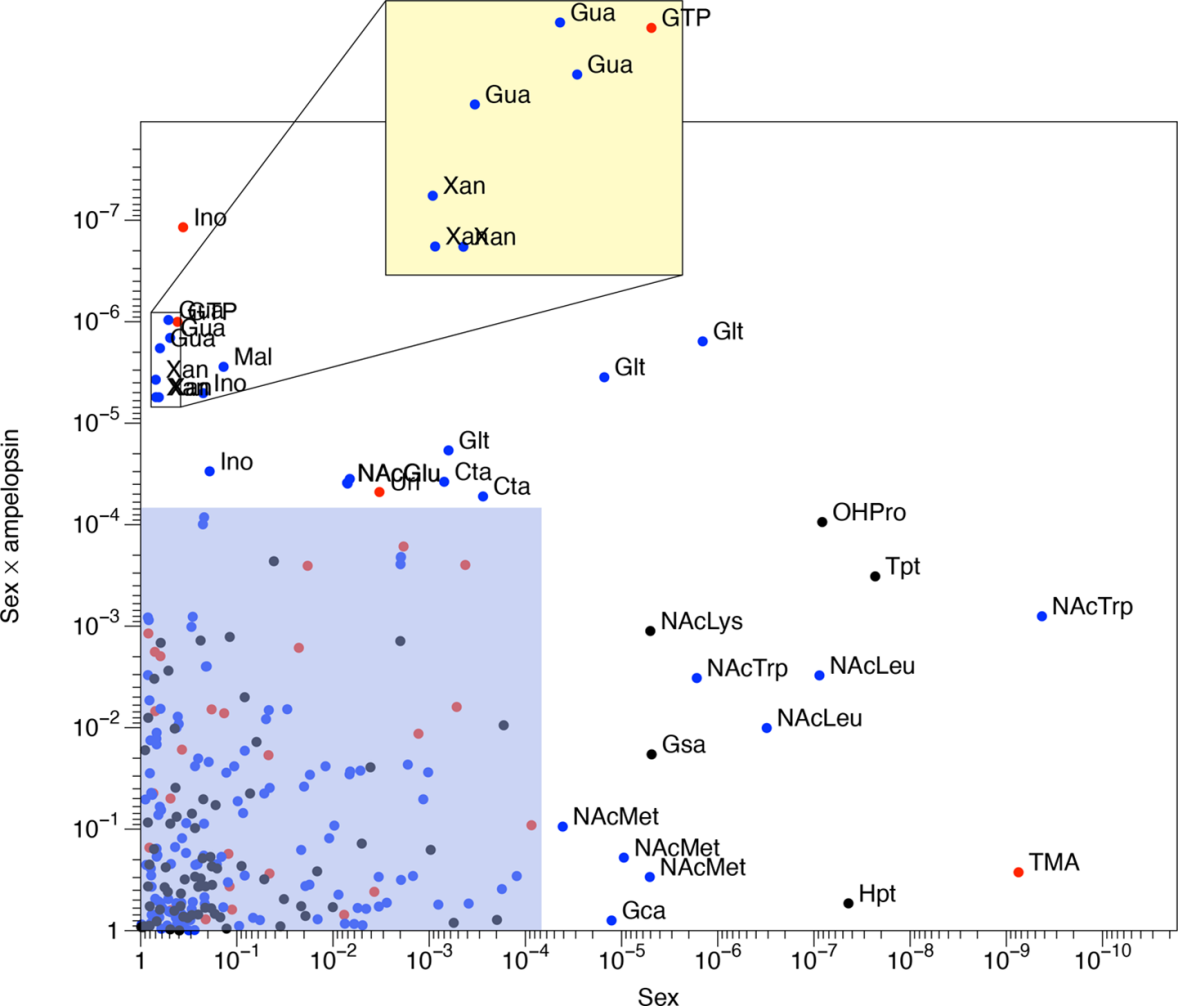
Metabolites differing between male and female mice tend to include *N*-acetylated amino acids, and metabolites with an interaction between sex and ampelopsin treatment tend to include organic acids and nucleosides. Data taken from two-way ANOVA. Black: AccQ-Tag RPLC data; blue: IPC data; red: ^1^H NMR data. Data points refer to metabolites (NMR) or transitions (LC-MS), such that a single metabolite may be represented by several data points. Blue shaded area indicates metabolites with *P* > 6.8 × 10^–5^ (i.e., corresponding to original *P* value threshold of 0.01 following Bonferroni correction). Yellow shaded area: magnification of crowded region of the plot. Metabolites are labeled directly on the plot. Glt: glutarate; NAcTrp: *N*-acetyltryptophan; NAcLeu: *N*-acetylleucine; NAcMet: *N*-acetylmethionine; Gca: gluconate; Tpt: tryptamine; Hpt: hypotaurine; OHPro: hydroxyproline; NAcLys: *N*α-acetyllysine; Gsa: glucosaminate; TMA: trimethylamine; Ino: inosine; Xan: xanthine; Gua: guanosine; GTP: guanosine triphosphate; Mal: malate; NAcGlu: *N*-acetylglutamate; Uri: uridine; Cta: cysteate.

When analyzing the data for the male and female mice separately, the metabolite with the most significant effect of ampelopsin treatment was inosine, in male mice, with *P* < 10^–5^ for both ^1^H NMR spectroscopic and IPC detection (Figure 4). Given the interaction shown by the PCA, it is not surprising that the fold-change of all metabolites with respect to ampelopsin treatment were negatively correlated (*r* = −0.60, P = 3.5 × 10^–27^, log-transformed data) between male and female mice (Figure 5); i.e., ampelopsin exerted opposite metabolic effects in male and female mice. This gives grounds for a further univariate analysis, as analysis of variance allows formal testing of the significance of the interaction. The results of this (Table S7, Supporting Information) show a number of significant metabolites. However, following Bonferroni correction (based on the number of metabolites, as this represents the underlying number of variables), no metabolites remained with a significant effect of ampelopsin treatment as a sole factor. There were a number of metabolites that had a significant effect of sex, particularly *N*-acetylated amino acids, and also a number that had a significant interaction between sex and ampelopsin treatment, including nucleosides and related compounds (uridine, guanosine, inosine, xanthine, GTP), and organic acids (cysteate, malate, *N*-acetylglutamate, and glutarate). Glutarate was the only compound that was significant with respect to both sex and to the interaction term sex × ampelopsin (Figure 5). The aging process and its health outcome differ in male and female mice (although mechanisms are still poorly understood), and so the metabolic differences found here are reasonable.^41^ Moreover, administration of trehalose in C57BL/6N old mice affects healthspan (behavior and brain antioxidant defenses) in a sex-dependent fashion, similarly suggesting the ability of natural compounds to target specific aspects of age- and sex-dependent vulnerability.^42^

Overall, the complementary combination of amine derivatization and ion-pairing chromatography described here provides a robust approach for targeted analysis of the polar metabolome. The methods could, of course, be improved further. Perhaps the most obvious improvement would be addition of more analytes to these two methods. It would also be possible to add in additional complementary analytical methods—for example, RPLC would be an obvious third method to include, in particular for those compounds that ionize poorly or not at all in negative mode, as the presence of tributylamine in the mobile phase makes positive mode ESI impractical for the IPC method. There is also, of course, scope to add in further targeted complementary analyses for subgroups of the polar metabolome (e.g., thiol metabolites would be an obvious choice, given their lability), but these will not be discussed further here. Although these are targeted methods, and meet Metabolomics Standards Initiative (MSI) level 1 identification criteria (i.e., metabolite assignments are based on *t*_R_, parent and daughter ion *m*/*z*, and comparison to authentic standards^43^), they still do not necessarily ensure compound identity beyond doubt when analyzing biological samples. Structural isomers are an obvious case where there is potential for misassignment: even if two isomers are resolved as pure standards, it may often be the case that only one of these isomers is present at detectable levels in biological samples. This can make it hard to assign compound identity with complete certainty, especially as there may often be slight *t*_R_ shifts when comparing chromatograms of pure standards to those derived from complex biological matrices. This is not a critical difficulty—peak assignments can generally be confirmed, if necessary, by spiking the authentic standard into the biological sample and reacquiring the data. (If even this is not sufficient, then we suggest that reacquiring with alternative chromatography, e.g., using a pentafluorophenyl-derivatized column, should provide enough data for unambiguous assignment.) The problem is particularly acute for the AccQ-Tag-derivatized metabolites: the daughter ion spectra tend to be dominated by the peak from the 6-aminoquinoline formyl ester fragment, generally to the extent that this is the only ion observed. Because of this, the amount of structural information is equivalent only to that conveyed by a single quadrupole when comparing between derivatized analytes, although of course signals from compounds which do not contain a derivatized amine group will still be filtered out. We still strongly think that these data are worth acquiring, despite the loss of structural information.

## Conclusion

Both amine derivatization and ion-pairing chromatography had significant benefits over standard reversed-phase chromatography for a wide range of metabolites: they both led to a valuable improvement in retention times for a significant proportion of the metabolome. Furthermore, they are naturally complementary: amine-containing compounds will tend to be ones that can take a positive charge, whereas the ion-pairing reagent used here (tributylamine) will associate with negatively charged analytes. When applying these methods to a real biological sample set—livers from mice that had been treated with a phytochemical—both the AccQ-Tag RPLC and IPC methods indicated similar overall metabolic patterns, but did so by identifying unique metabolic changes, i.e., metabolite biomarkers that would have been missed if only one of the methods had been employed. Using both of these approaches together provides a robust platform that covers a large proportion of the metabolome, and thus would be widely applicable in metabolomic research. While these methods for accessing the polar metabolome are not a panacea, and do not replace other approaches such as, e.g., HILIC, we believe that they do provide useful complementary approaches.

## Supporting Information Available

Figure S1. Representative peak shape for ion pairing chromatography; Figure S2. Comparison of retention times for metabolites with two different ion-pairing reagents; Figure S3. Comparison of sensitivity for RPLC-MS vs IPLC-MS; Figure S4. Plot of CV_biol_/CV_QC_ against CV_QC_; Figure S5. Correlations between NMR, AccQ-Tag, and ion-pairing data for 2-aminoadipate; Table S1. LC-MS settings for all methods (PDF)
Table S2. Retention time data (XLSX)
Table S3. MS parameters for metabolites monitored in this study (XLSX)
Table S4. Metabolite data from LC-MS (AccQ-Tag RPLC) (XLSX)
Table S5. Metabolite data from ^1^H NMR spectroscopy (XLSX)
Table S6. Metabolite data from LC-MS (IPC) (XLSX)
Table S7. Output of statistical analysis of liver data (XLSX)
