# Supplementary material for: Ion-Pairing Chromatography and Amine Derivatization Provide Complementary Approaches for the Targeted LC-MS Analysis of the Polar Metabolome
Source: J Proteome Res. 2022 May 10;21(6):1428–37. doi: 10.1021/acs.jproteome.2c00030 (PMC9171846; doi:10.1021/acs.jproteome.2c00030)
Supplement: Supplementary file 1 — pr2c00030_si_001.pdf [file pr2c00030_si_001.pdf]

Ion-pairing chromatography and amine derivatization provide complementary approaches for the targeted LC-MS analysis of the polar metabolome

Virag Sagi-Kiss,[1] Yufeng Li,[1] Matthew R. Carey,[1] Sarah J. Grover,[1] Karsten Siems,[2] Francesca Cirulli,[3] Alessandra Berry,[3] Chiara Musillo,[3,4] Ian D. Wilson,[1] Elizabeth J. Want,[1] Jacob G. Bundy*[1]

1: Department of Metabolism, Digestion and Reproduction, Imperial College London, South Kensington, London SW7 2AZ, UK

2: Analyticon Discovery GmbH, Hermannswerder Haus 17, 14473 Potsdam, Germany

3: Center for Behavioral Sciences and Mental Health, Istituto Superiore di Sanità, Rome, Italy

4: Department of Psychology, Sapienza University of Rome, Italy

Table of Contents

Table S1. Settings used for liquid chromatography-mass spectrometry experiments.

Table S2 (XLSX). Retention time data.

Table S3 (XLSX). MS settings for metabolites monitored by final combined method.

Table S4 (XLSX). Metabolite data from LC-MS (AccQ-Tag RPLC).

Table S5 (XLSX). Metabolite data from ^{1}H NMR spectroscopy.

Table S6 (XLSX). Metabolite data from LC-MS (IPC).

Table S7 (XLSX). Output of statistical analyses for liver samples.

Figure S1. Exemplar chromatograms for ion-pairing chromatography. Some metabolites exhibit sharp, Gaussian peaks; others have poorer peak shape, but can still be analysed if the peaks are well resolved from others.

Figure S2. Comparison of retention times for metabolites with two different ion-pairing reagents, tributylamine (TBA) + acetic acid, vs. diisopropylethylamine (DIPEA) + hexafluoroisopropanol. Metabolites of different chemical classes are indicated on the plot.

Figure S3. Comparison of sensitivity for RPLC-MS vs. IPLC-MS (n=3, ± SD).

Figure S4. Plot of CV_{biol}/CV_{QC} against CV_{QC} for data derived from ion-pairing LC-MS of mouse liver extracts. There is an apparent step in the data at approximately $CV_{QC} = 0.5$. Individual data points represent transitions, rather than metabolites.

Figure S5. Correlations between NMR, AccQ-Tag, and ion-pairing data (for three different transitions) for 2-aminoadipate.

Table S1. Settings used for liquid chromatography-mass spectrometry experiments.

	AccQ-Tag RPLC	RPLC POS	IPC	RPLC NEG
LC unit	ACQUITY Binary Solvent Manager (Waters Corporation) + CTC Pal autosampler	UFLX XR (Shimadzu)	ACQUITY Binary Solvent Manager (Waters Corporation) + CTC Pal autosampler	ACQUITY Binary Solvent Manager (Waters Corporation) + CTC Pal autosampler
Mobile phase A	0.1 % Formic acid water	0.1 % Formic acid water	10mM of tributylamine + 15mM of acetic acid + acetylacetone (0.02% v/v) in water	0.1 % Formic acid water
Mobile phase B	0.1 % Formic acid Acetonitrile	0.1 % Formic acid Acetonitrile	80% methanol and 20% isopropanol	0.1 % Formic acid Acetonitrile
Flow rate	0.6 ml/min	0.4 ml/min	0.4 ml/min	0.4 ml/min
Column temperature	50 ℃	45 ℃	45 ℃	45 ℃
Column	Waters HSS T3 (1.8 µm, 2.1 x 150 mm)	Waters HSS T3 (1.8 µm, 2.1 x 100 mm)	Waters HSS T3 (1.8 µm, 2.1 x 100 mm)	Waters HSS T3 (1.8 µm, 2.1 x 100 mm)
Injection volume	5 µl	5 µl	5 µl	5 µl
Gradient	0.1 to 12 min from 1% solvent B to 28% solvent B; at a flow rate of 0.6 mL/min. Post-acquisition wash and recovery steps took 2.65 min in total and went up to 100% B, and 1 min at initial conditions for equilibration.	0min 1%; 0.5 min, 1% B; 10 min, 55% B; 11 min, 100% B; 12 min, 100% B; 12.5 min, 1% B; 14.5 min, 1% B.	0-0.5 min: 0% B; 0.5-6 min: 0% B-5%B; 4-6min: 5%B; 6-6.5 min: 5-20% B; 6.5-8.5 min: 20% B; 8.5-14min: 20-55%B; 14-15 min: 55-100% B; 15-17min: 100%B; 17-18min 100-0% B. 3 min at initial conditions before next injection cycle starts.	0min: 99% A; 0.5min: 99%A; 10 min: 45%A; 11min: 0%A; 12 min: 0%A; 12.5min: 99%A; 14.5 min: 99%A. Linear gradient between all times
MS type	Waters XEVO TQ-S (Waters Corporation)	QTRAP 5500 MS/MS system [Applied Biosystem (CA, USA)]	Waters XEVO TQ-S (Waters Corporation)	Waters XEVO TQ-S (Waters Corporation)
Ion mode	POS	POS	NEG	NEG
Source parameters	capillary voltage, 1.5 kV; source temperature, 150°C; cone voltage: 40 V;	De-clustering, entrance and collision cell exit potentials were set to 30 V, 10 V and 10 V, respectively.	capillary voltage, 1.5 kV; source temperature, 150°C; cone voltage: 40 V;	capillary voltage, 2 kV; source temperature, 150°C; cone voltage: 40 V;
AdditIonal source settings:	desolvation temperature 600°C; desolvation gas flow, 1000 L/h; cone gas flow, 150 L/h; nebuliser gas 7.0 bar; collision gas, 0.15 mL/min.	Curtain gas, 40 psi; collision gas, medium; ionspray voltage, 5500 V; temperature, 550°C; ion source gas 1 and 2, 40 and 60 psi, respectively.	desolvation temperature 600°C; desolvation gas flow, 1000 L/h; cone gas flow, 150 L/h; nebuliser gas 7.0 bar; collision gas, 0.15 mL/min.	desolvation temperature 300°C; desolvation gas flow, 1000 L/h; cone gas flow, 150 L/h; nebuliser gas 7.0 bar; collision gas, 0.15 mL/min.

Figure S1.

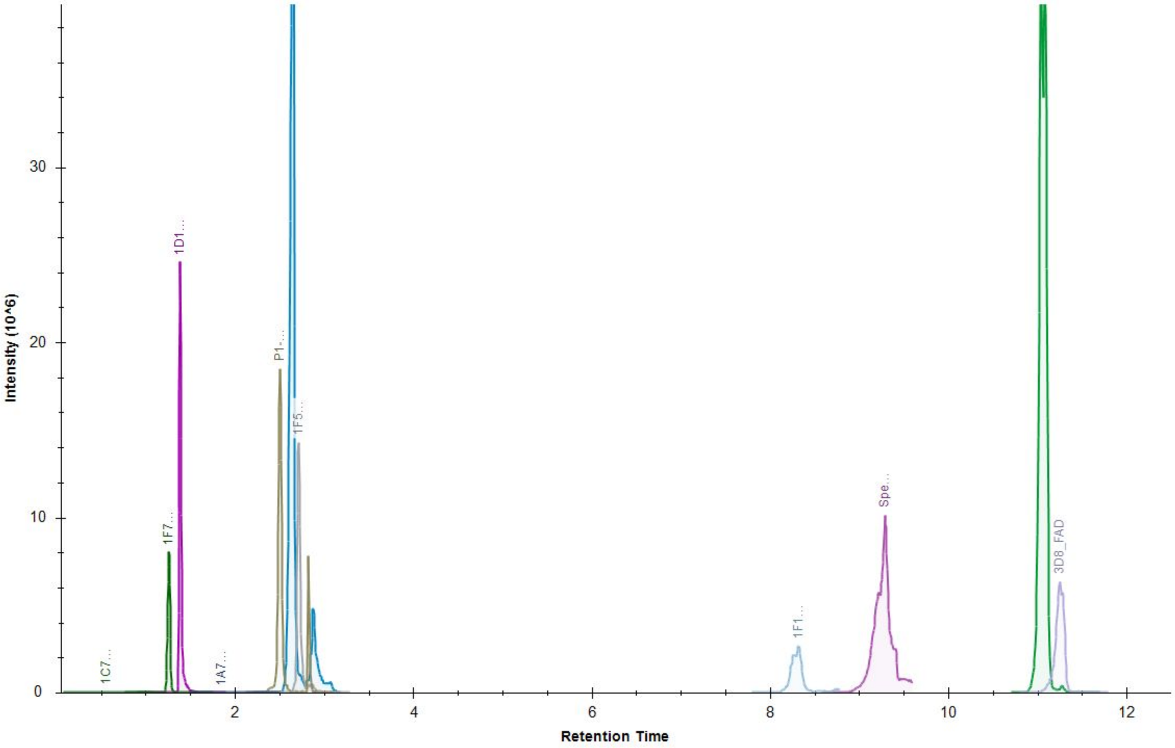

Figure S1. Exemplar chromatograms for ion-pairing chromatography. Some metabolites exhibit sharp, Gaussian peaks; others have poorer peak shape, but can still be analysed if the peaks are well resolved from others.

Figure S2

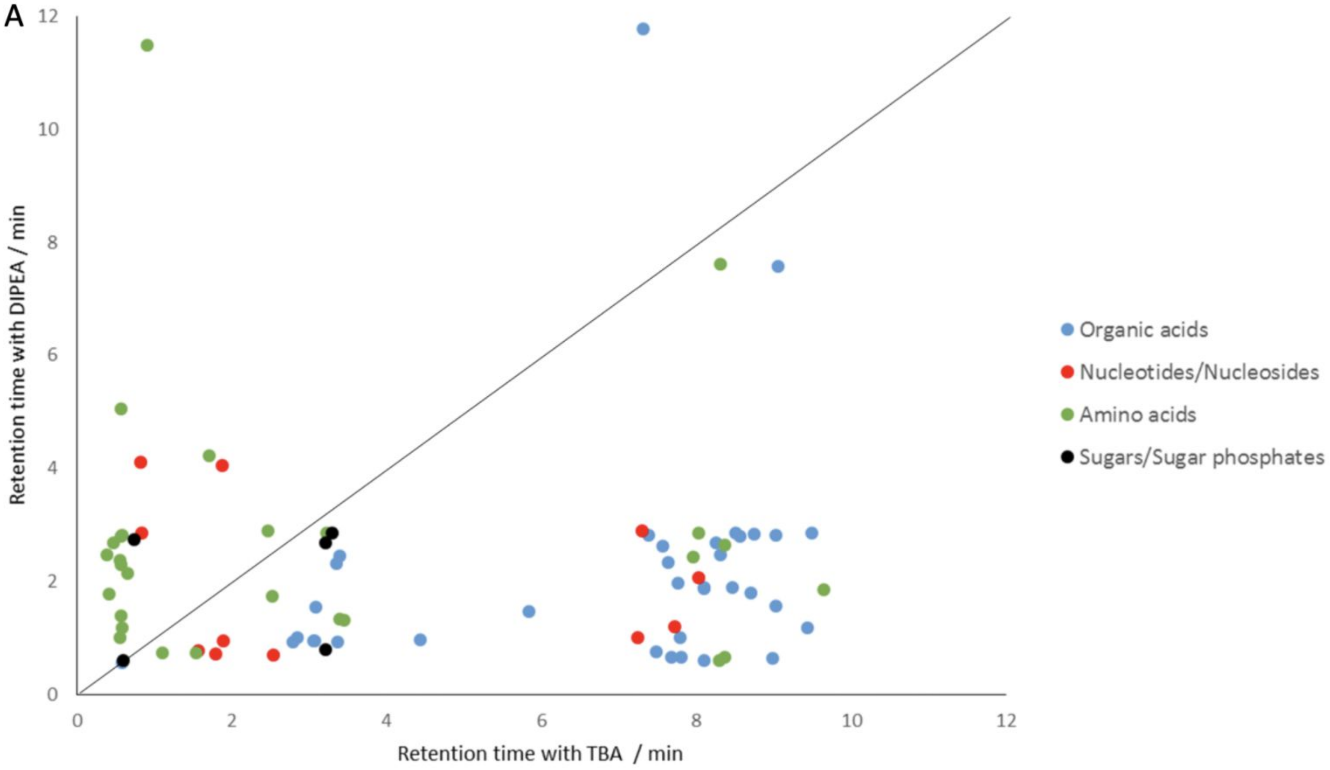

Figure S2. Comparison of retention times for metabolites with two different ion-pairing reagents, tributylamine (TBA) + acetic acid, vs. diisopropylethylamine (DIPEA) + hexafluoroisopropanol. Metabolites of different chemical classes are indicated on the plot.

Figure S3

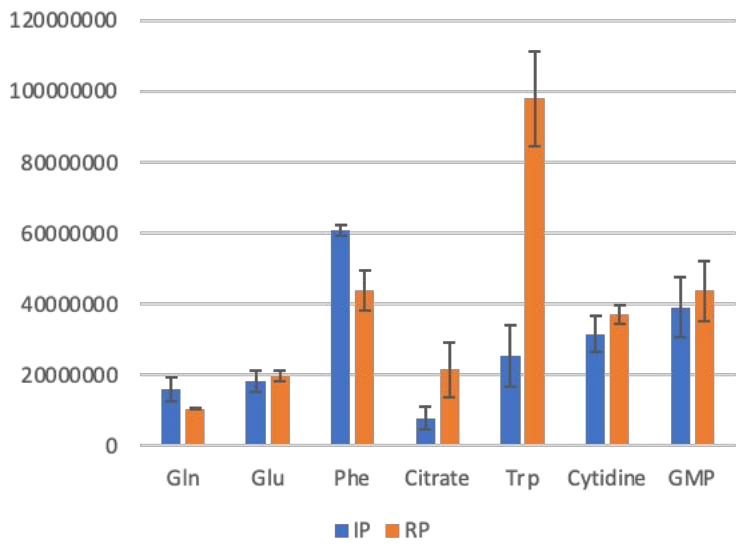

Figure S3. Comparison of sensitivity for RPLC-MS vs. IPLC-MS (n=3, ± SD).

Figure S4

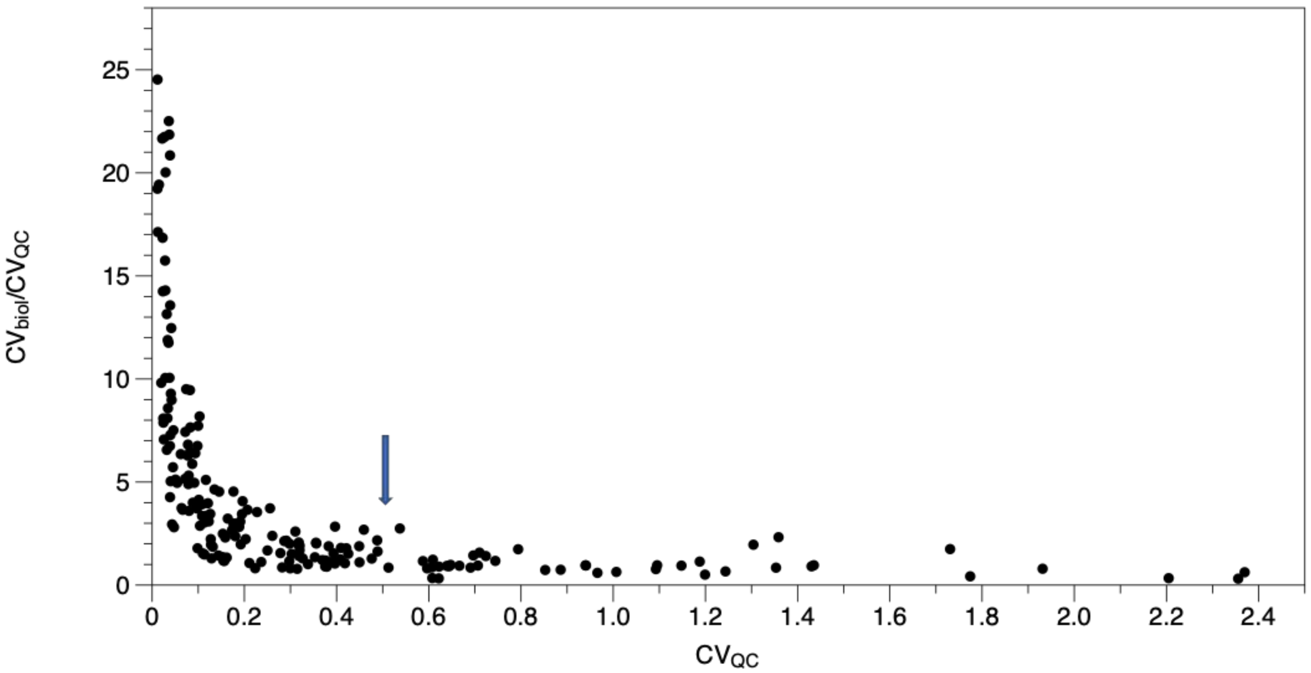

Figure S4. Plot of CV_{biol}/CV_{QC} against CV_{QC} for data derived from ion-pairing LC-MS of mouse liver extracts. There is an apparent step in the data at approximately $CV_{QC} = 0.5$. Individual data points represent transitions, rather than metabolites.

Figure S5

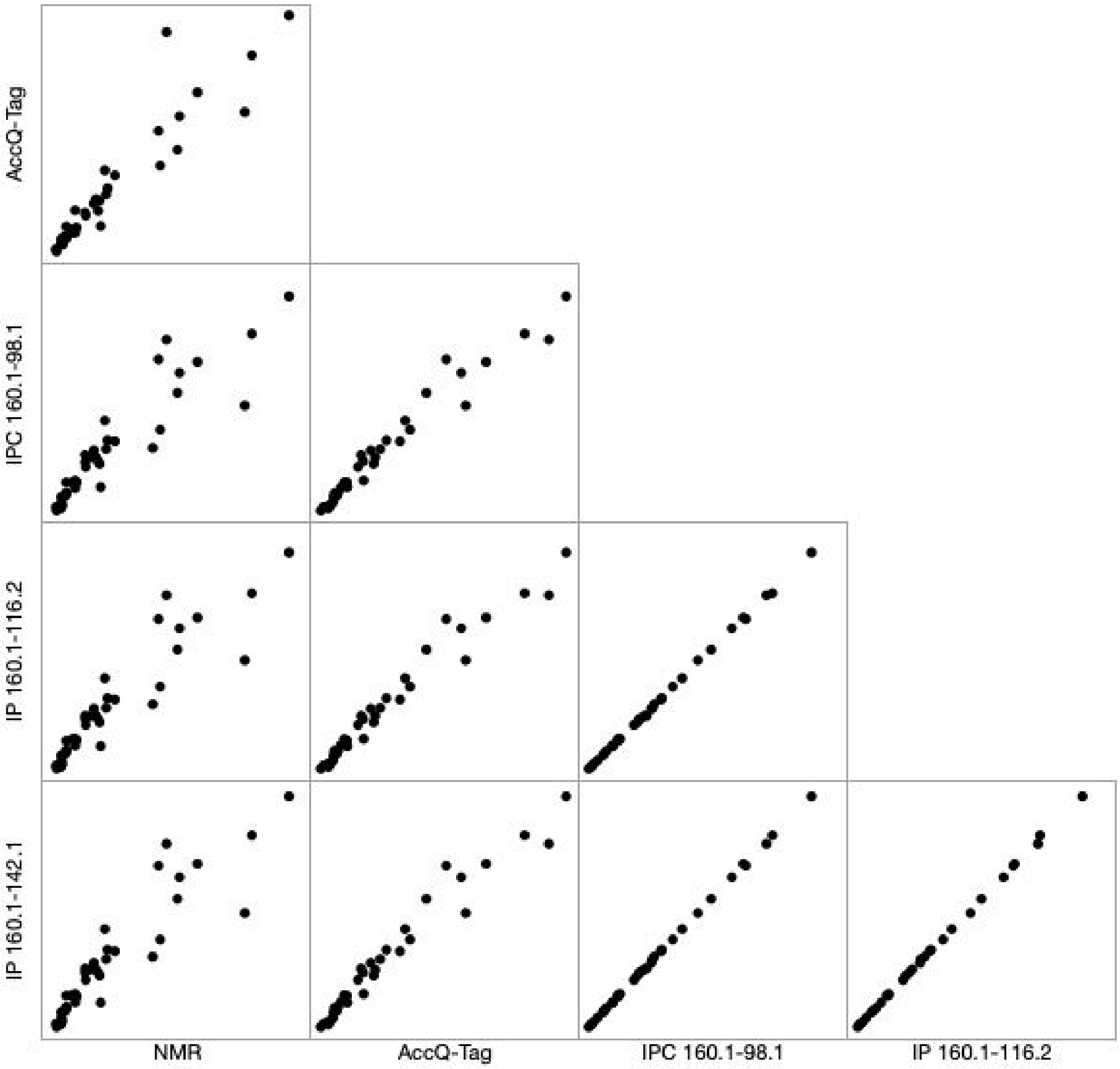

Figure S5. Correlations between NMR, AccQ-Tag, and ion-pairing data (for three different transitions) for 2-aminoadipate.
